# Modeling, Simulation, and Reconstruction of 2-Reservoir Heat-to-Power Processes in Finite-Time Thermodynamics †

**DOI:** 10.3390/e22090997

**Published:** 2020-09-07

**Authors:** Wolfgang Muschik, Karl Heinz Hoffmann

**Affiliations:** 1 Institut für Theoretische Physik, Technische Universität Berlin, Hardenbergstr. 36, 10623 Berlin, Germany; 2 Institut für Physik, Technische Universität Chemnitz, 09107 Chemnitz, Germany; hoffmann@physik.tu-chemnitz.de

**Keywords:** simulation, modeling, reconstruction, finite time thermodynamics, endoreversible thermodynamics, nonequilibrium thermodynamics, entropy production, contact temperature

## Abstract

The connection between endoreversible models of Finite-Time Thermodynamics and the corresponding real running irreversible processes is investigated by introducing two concepts which complement each other: *Simulation* and *Reconstruction*. In that context, the importance of particular machine diagrams for *Simulation* and (reconstruction) parameter diagrams for *Reconstruction* is emphasized. Additionally, the treatment of internal irreversibilities through the use of contact quantities like the contact temperature is introduced into the Finite-Time Thermodynamics description of thermal processes.

## 1. Introduction

“Finite-Time Thermodynamics” (FTT) is a field in nonequilibrium thermodynamics that evolved over the past 45 years and that has, and still does, attract a lot of interest. Its central theme is that finishing a thermodynamic task in a finite amount of time is different from doing it with an infinite time horizon. If only limited time is available for a cyclic process to convert heat into work with a macroscopic heat engine, then one usually has to pay a “price” in form of a reduced efficiency. Finite-Time Thermodynamics set out to quantify that price.

This theme has been investigated in a vast variety of paradigmatic examples. If one wants to stress the fact that nonequilibrium processes will have performance features different from an equilibrium description, then one has to make that point for the present with the help of simple examples and not with the complexity of a real running heat engine like, for instance, a jet turbine. Such a simple paradigmatic example is the Curzon–Ahlborn efficiency [1] for a maximum power heat-to-work conversion:

(1) $$\begin{array}{c} \eta_{\mathrm{CA}}=1-\sqrt{\frac{T_{L}}{T_{H}}}, \end{array}$$

where $T_{L}$ and $T_{H}$ are, respectively, the temperatures of the low- and high-temperature heat baths a Carnot engine is operating between under the restriction that the heat flows to and from the engine are limited by a finite heat conduction. The idea in that example is not to predict the efficiencies of real running power stations for a quantitative analysis, but to show that the Curzon–Ahlborn efficiency is a much better predictor for observed efficiencies than the Carnot efficiency,

(2) $$\begin{array}{c} \eta_{\mathrm{Carnot}}=1-\frac{T_{L}}{T_{H}}. \end{array}$$

Later, it became clear that already earlier Novikov [2] and others [3] had used an even simpler model by considering only one heat flow to be restricted by a limited heat conductance, while the the other flow is reversible. Nonetheless, the Curzon–Ahlborn efficiency also applies to these models.

Finite-Time Thermodynamics evolved over the years and different aspects of nonequilibrium processes were analyzed. Early work started in Steve Berry’s group [4,5,6,7], and later the field evolved into different directions, for a review see in [8]. While originally the focus was on macroscopic heat engines and their optimization [9,10,11,12], lately also quantum engines have attracted interest [13,14,15]. Again, the goal is to find performance extrema of heat to power conversion [16,17], but also generalizations of the classical availability concept to the quantum level have been considered [18,19].

Finite-Time Thermodynamics as a field is open for different methods, but always with goal to capture the impact of “haste” in performing a thermodynamic process. This is for instance apparent in the work on finite-time potentials [5] or, more recently, on the implementation of finite-time concepts in the realm of biological processes [20]. When it comes to quantifying the necessary irreversibility with its performance losses due to “haste”, Endoreversible Thermodynamics [21,22,23,24,25,26,27] has shown its great potential as a modeling tool. Its basic concept is to describe a system undergoing nonequilibrium processes as consisting of reservoirs, engines, and reactors, which are modeled as reversible systems, such that the usual thermodynamic equilibrium relations apply. All dissipation is confined to the interactions between those systems, which capture the nonequilibrium transport of energy and other thermodynamic extensities. Usually these are characterized by transport equations for the irreversible processes, which contain characteristic and often fixed parameters like a heat conduction or a flow viscosity. Endoreversible Thermodynamics has been used, for instance, in the treatment of heat-to-power conversion [28,29,30,31,32,33], in the context of chemical processes [34,35], in thermo-economic applications, [36,37] and in the thermodynamics of computing [38].

In this paper two advancements beyond FTT are presented: The first one goes beyond the limitations of Endoreversible Thermodynamics, following from the assumption of endoreversibility for the subsystems in question. While Endoreversible Thermodynamics uses the fact that it treats subsystems as reversible without internal entropy production, here it is demonstrated that the use of nonequilibrium quantities like contact temperature for heat flows or nonequilibrium molar entropies for material flows allows to include internal irreversibilities for describing nonequilibrium states appropriately. The second advancement is to go beyond the use of paradigmatic but simple models. It thus sheds light on the modeling character of endoreversible systems in relation to real running heat engines. To elucidate the different perspectives taken—on the one hand, starting from a model and, on the other hand, starting from the performance of *real running* heat engine—the concepts of *simulation* and *reconstruction* are introduced. For the presentation of both advancements, two simple and well-known cyclic 2-reservoir heat-to-power model processes are chosen.

The paper is organized as follows. After this introduction, the nonequilibrium time rate of discrete (Schottky) systems is repeated for elucidation of the sequel and for defining a nonequilibrium temperature—the contact temperature—in the third section. The contact temperature is essential for describing real running (irreversible) cyclic 2-reservoir heat-to-power processes in the fourth section. Because contact and reservoir temperatures are used side by side, two different entropy productions appear which are connected by a function of the net heat flows and the contact and reservoir (baths) temperatures—the *non-reversibility.* In the fifth section, a process class is introduced by which the tools of simulation and modeling are defined. These are then applied to two historical basic endoreversible models of FTT: the reversible Carnot process with heat leak and the Curzon–Ahlborn model, both subjected to a simulation of a real running process and conversely subjected to a reconstruction of a model of an engine.

## 2. Entropy Time Rate of Discrete Systems

### 2.1. Equilibrium

A discrete system (also named Schottky system [39]) $G^{*}$ in equilibrium is considered which is presupposed to be a reservoir. This implies that the relaxation times of the system are arbitrarily high and that $G^{*}$ can be described as being always in equilibrium. Consequently, $G^{*}$ is subjected to thermostatics whose validity is presupposed. The *“time rate” of entropy* of the reservoir is

(3) $$\overset{{}_{{}^{•}}}{S}{}^{*}\;=\;\frac{1}{T^{*}}\overset{{}_{{}^{•}}}{Q}{}^{*}+s{}^{*}\cdot \overset{{}_{{}^{•}}}{n}{\;}^{*e},$$

and the differentials of thermostatics are written as derivatives

(4) $$d\;⊕\;\equiv \;\overset{{}_{{}^{•}}}{⊕}$$

because of adapting the formalism to nonequilibrium in the sequel (more details in [40]). The entropy flux in (3) is a factorized decomposition into the reciprocal thermostatic temperature $T^{*}$ of $G^{*}$ and the heat exchange through its surface $\partial G^{*}$. Moreover, the components of the external material exchange $\overset{{}_{{}^{•}}}{n}{\;}^{*e}$ are in reference to $G^{*}$. The molar entropies of the components in $G^{*}$ are $s{}^{*}$. An entropy production does not appear in (3), because $G^{*}$ is an equilibrium system and consequently described by thermostatics.

### 2.2. Non-Equilibrium, 2nd Law and Compound Systems

The *time rate of entropy* of a system G in nonequilibrium has the form (more details in [40])

(5) $$\overset{{}_{{}^{•}}}{S}\;=\;\frac{1}{\Theta }\overset{{}_{{}^{•}}}{Q}+s\cdot \overset{{}_{{}^{•}}}{n}{}^{e}+\Sigma .$$

Here, $\overset{{}_{{}^{•}}}{Q}$ and $\overset{{}_{{}^{•}}}{n}{}^{e}$ are the heat exchange and the external material exchange through the surface ∂G of G. The thermostatic temperature $T^{*}$ in (3) as well as the equilibrium molar entropies $s^{*}$ have to be replaced by nonequilibrium quantities, contact temperature, Θ and nonequilibrium molar entropies *s*, which are defined in the sequel. The entropy production Σ is independent of the exchange quantities $\overset{{}_{{}^{•}}}{Q}$ and $\overset{{}_{{}^{•}}}{n}{}^{e}$, and consequently, Σ is the time rate of entropy in isolated systems ($\overset{{}_{{}^{•}}}{Q}\equiv 0$ and $\overset{{}_{{}^{•}}}{n}{}^{e}\equiv 0$).

According to the *Second Law*, the entropy production is not negative [41,42,43] (a statement which is in such a way not valid in Stochastic Thermodynamics [44]),

(6) Σ≥0.

A comparison of (5) with (3) shows that the entropy production $\Sigma^{*}\equiv 0$ vanishes identically in equilibrium systems.

Now, a nonequilibrium system G is considered which is embedded into an equilibrium reservoir $G^{*}$ having a joint surface $\partial G\equiv \partial G^{*}$, which means a compound system $G\cup G^{*}$ is considered whose sub-systems have mutual exchanges of heat and material. Usually, $G^{*}$ is denoted as the system’s controlling environment. The joint surface represents the partition between the two subsystems. Especially, inert partitions are considered which are defined as follows. An inert partition does not absorb or emit heat, power, and material [45], as described by the following equations [46,47],

(7) $$\overset{{}_{{}^{•}}}{Q}\;=\;-\overset{{}_{{}^{•}}}{Q}{}^{*},\;W\;=\;A\cdot \overset{{}_{{}^{•}}}{a}\;=\;A^{*}\cdot \overset{{}_{{}^{•}}}{a}\;=\;-W^{*},\;\overset{{}_{{}^{•}}}{n}{}^{e}\;=\;-\overset{{}_{{}^{•}}}{n}\;{}^{*e}.$$

Here, the ${}^{*}$-quantities belong to the system’s controlling environment $G^{*}$. The work done on the system is performed by the environment using its generalized forces $A^{*}$ and orientated at the work variables of the system (which do not appear according to (3) and (4)). The permeability of ∂G to heat, power, and material is described by (7). The time rate of entropy of the compound system is set by an

Axiom: The partial entropies of sub-systems are additive.

The entropy of the isolated total system $G^{*}\cup G$ is according to this axiom and (7)${}_{1,3}$ (a subscript *i* of an equation reference refers to the *i*th (in)equalitiy or *i*th element in the referenced equation; here, (7)${}_{1,3}$ refers to the first and last equation in (7))

(8) $$\begin{array}{ccc} \overset{{}_{{}^{•}}}{S}{\;}^{tot}\;:=\;\overset{{}_{{}^{•}}}{S}+\overset{{}_{{}^{•}}}{S}\;{}^{*} & = & \frac{1}{\Theta }\overset{{}_{{}^{•}}}{Q}+s\cdot \overset{{}_{{}^{•}}}{n}\;{}^{e}+\frac{1}{T^{*}}\overset{{}_{{}^{•}}}{Q}\;{}^{*}+s{}^{*}\cdot \overset{{}_{{}^{•}}}{n}{\;}^{*e}+\Sigma \;=\;\\ & = & \left(\frac{1}{\Theta }-\frac{1}{T^{*}}\right)\overset{{}_{{}^{•}}}{Q}+\left(s-s{}^{*}\right)\cdot \overset{{}_{{}^{•}}}{n}\;{}^{e}+\Sigma \;\geq \;0. \end{array}$$

The inequality sign is due to the isolation of the compound system and the definition of entropy production. The inequality (8)${}_{3}$ allows to define the contact temperature Θ and the nonequilibrium molar entropies ***s*** in Section 3. Now, another property of the time rate of nonequilibrium entropy (5) is considered.

### 2.3. Non-Equilibrium Entropy as a State Function

For defining the time rate of nonequilibrium entropy, a *state space* Z for G is needed,

(9) $$\overset{{}_{{}^{•}}}{S}\left(Z\left(t\right)\right)\;=\;\frac{\partial S}{\partial Z}\cdot \overset{{}_{{}^{•}}}{Z}\left(t\right),\;Z\in Z.$$

Such a nonequilibrium state space is spanned by the equilibrium variables internal energy *U*, the work variables ***a*** and the mol numbers ***n*** of the system, supplemented by the nonequilibrium variables contact temperature Θ and the internal variables ξ [48,49]

(10) Z=(a,n,U,Θ,ξ)∈Z.

The choice of such a state space is possible, because in nonequilibrium *U* and Θ are independent variables, and the entropy production depends on the time rates of the nonequilibrium variables $\Sigma (\overset{{}_{{}^{•}}}{\Theta },\overset{{}_{{}^{•}}}{\xi })$ [50].

The time rate of the nonequilibrium entropy has to be in accordance with the equilibrium entropy. This fact is enforced by the embedding theorem: the nonequilibrium entropy rate integrated along an irreversible process T starting and ending in equilibrium states—$A_{eq}$ and $B_{eq}$—has the same value as the difference of the equilibrium entropies between the initial and final states of T,

(11) $$T\int_{A_{eq}}^{B_{eq}}\overset{{}_{{}^{•}}}{S}\left(Z\left(t\right)\right)dt\;=\;S\left(B_{eq}\right)-S\left(A_{eq}\right).$$

Beyond the embedding theorem, an other property is necessary for establishing a nonequilibrium entropy to be a state function on Z: adiabatical uniqueness defined as follows [45].

**Definition** **1.** *A Schottky system is called* adiabatically unique, *if, for each arbitrary but fixed nonequilibrium state B, after isolation of the system the relaxation process ends always in the same final equilibrium state, independently of how the process into B was performed.*

Considering a cyclic process in Z, taking into account (6) and that S(Z) is a state function on Z, (5) results in

(12) $$\oint \overset{{}_{{}^{•}}}{S}dt\;=\;0\;=\;\oint \left(\frac{1}{\Theta }\overset{{}_{{}^{•}}}{Q}+s\cdot \overset{{}_{{}^{•}}}{n}\;{}^{e}\right)dt+\oint \Sigma dt\;\geq \;\oint \left(\frac{1}{\Theta }\overset{{}_{{}^{•}}}{Q}+s\cdot \overset{{}_{{}^{•}}}{n}\;{}^{e}\right)dt.$$

Consequently, the entropy production of a cyclic process becomes according to (12)${}_{2}$ and (6)

(13) $$\oint \Sigma dt\;=\;-\oint \left(\frac{1}{\Theta }\overset{{}_{{}^{•}}}{Q}+s\cdot \overset{{}_{{}^{•}}}{n}\;{}^{e}\right)dt\;\geq \;0.$$

The definitions of the contact temperature Θ and the nonequilibrium molar entropy ***s*** which appear in (8)${}_{2}$ and in the Clausius-like inequality (13) are given in the next section.

## 3. Contact Temperature and Neq-Molar Entropy

Up to now, Θ and ***s*** are placeholders in the dissipation inequality (8)${}_{2}$ for the unknown contact quantities, whereas Σ, the internal entropy production of the system according to (5), is represented by $\left(1/\Theta -1/T^{*}\right)\overset{{}_{{}^{•}}}{Q}$ and $\left(s-s{}^{*}\right)\cdot \overset{{}_{{}^{•}}}{n}\;{}^{e}$ is the entropy production of the heat and material exchanges between the subsystems of the compound system. If the system is a reversible one (Σ=0), these exchanges have to be compatible with the dissipation inequality (8)${}_{2}$. Because heat and material exchanges are independent of each other, the following inequalities

(14) $$\left(\frac{1}{\Theta }-\frac{1}{T^{*}}\right)\overset{{}_{{}^{•}}}{Q}\;\geq \;0\;\left(s-s{}^{*}\right)\cdot \overset{{}_{{}^{•}}}{n}\;{}^{e}\;\geq \;0$$

are demanded for defining the placeholders Θ and ***s*** which are ascribed to the subsystem G (the system) of the compound system $G^{*}\cup G$.

For defining these place holders, the following proposition [51] for a vector quantity is used:

(15) X·f(X)≥0(for allX∧fcontinuous atX=0)⟹f(0)=0.

Without any restriction of generality, the left hand brackets in (14) can be presupposed as being continuous, if the right hand factors vanish. These factors vanish, if suitable equilibrium environments $G^{*}$ are chosen for contacting

(16) $$G_{⊙}^{*}\;⟶\;\overset{{}_{{}^{•}}}{Q}{\;}_{⊙}=0,\;G_{j0}^{*}\;⟶\;\overset{{}_{{}^{•}}}{n}{\;}_{j0}^{e}=0,\;j=1,2,\ldots ,N\;\mathrm{components}.$$

$G_{⊙}^{*}$ and $G_{j0}^{*}$ are equipped with equal temperatures $T_{⊙}^{*}$ and $T_{0}^{*}$ which is the same for all $G_{j0}^{*}$. Consequently, according to the proposition (15) contact quantities can be defined, a temperature Θ and *N* molar entropies *s*, which belong to the special chosen environments $G_{⊙}^{*}$ and $G_{j0}^{*}$:

(17) $$\overset{{}_{{}^{•}}}{Q}{\;}_{⊙}=0\;⟺\;\Theta =T_{⊙}^{*},\;\overset{{}_{{}^{•}}}{n}{\;}_{0}^{e}=0\;⟺\;s=s_{0}^{*}.$$

Here, (17)${}_{2}$ holds true for each chemical component. The $T_{⊙}^{*}$ and $s_{0}^{*}$ are known and belong to the special equilibrium environments (16) which generate the vanishing RHS factors of (14). According to (17)${}_{1}$, the following definition is made [52,53,54]

**Definition** **2.** *The system’s* contact temperature Θ *is that thermostatic temperature* $T_{⊙}^{*}$ *of the system’s equilibrium environment for which the net heat exchange* $\overset{{}_{{}^{•}}}{Q}{\;}_{⊙}$ *between the system and this environment through an inert partition vanishes by change of sign.*

Inserting the defining inequalities (14) into the expression (12)${}_{3}$ for cyclic processes results in

(18) $$0\;\geq \;\oint \left(\frac{1}{\Theta }\overset{{}_{{}^{•}}}{Q}+s\cdot \overset{{}_{{}^{•}}}{n}{}^{e}\right)dt\;\geq \;\oint \left(\frac{1}{T^{*}}\overset{{}_{{}^{•}}}{Q}+s^{*}\cdot \overset{{}_{{}^{•}}}{n}{}^{e}\right)dt,$$

representing a proof and an extension of Clausius inequality: the thermostatic temperature $T^{*}$ and the molar entropies $s^{*}$ of the controlling equilibrium reservoirs which enforce the cyclic process are replaced by nonequilibrium quantities of the system, the contact temperature Θ, and the nonequilibrium molar entropies ***s***. Because the inequalities (6) and (14) change into equalities in equilibrium, the entropy rate in equilibrium is (3)

(19) $$\overset{{}_{{}^{•}}}{S}{}^{eq}\;=\;\frac{1}{T^{*}}\overset{{}_{{}^{•}}}{Q}+s^{*}\cdot \overset{{}_{{}^{•}}}{n}\;{}^{e},$$

which is a state function (or a total differential) on the equilibrium sub-space [55]

(20) $$Z^{eq}\;=\;\left(a,n,U,\Theta \left(a,n,U\right),\xi \left(a,n,U\right)\right)\;\in Z^{eq}\subset Z.$$

Even if the entropy production is added to the equilibrium entropy rate

(21) $$\overset{{}_{{}^{•}}}{S}{}^{eq}+\Sigma \;=\;\frac{1}{T^{*}}\overset{{}_{{}^{•}}}{Q}+s^{*}\cdot \overset{{}_{{}^{•}}}{n}\;{}^{e}+\Sigma \;\neq \;\overset{{}_{{}^{•}}}{S},$$

a comparison with (5) demonstrates that this expression is different from the nonequilibrium entropy rate. Consequently, it is not a state function because of the reservoir quantities $T^{*}$ and $s^{*}$ which do not belong to the system.

The utility of the contact quantities from a conceptual point of view is obvious. Their usage acknowledges the fact that real systems exchanging heat and work are not in equilibrium, and thus the assumption of endoreversibility is thus certainly not correct in the strict sense. From a practical point of view, the difference between a nonequilibrium contact temperature and an equilibrium temperature as a proxy in an overall description of thermodynamic systems depends very much on the “nonequilibrium” nature of the situation in question: In some cases the usage of an equilibrium proxy might be possible without much loss of accuracy, in other cases, for instance, when the local temperature field at the inert partition is highly nonuniform or if the assumption of local equilibrium no longer applies, the errors might be considerable. If in particular cases the contact temperature can be obtained in terms of the variables of a nonequilibrium state space, then also from a practical point of view their utility is even larger.

The short sketch of nonequilibrium thermodynamics given here uses explicitly the time and therefore includes Finite-Time Thermodynamics, which deals with irreversible cyclic processes in Schottky systems which are considered in the next sections.

## 4. Cyclic 2-Reservoir Processes

### 4.1. First Law

We consider two heat reservoirs (*H* and *L*) of different thermostatic (equilibrium) temperatures $T_{H}>T_{L}$. A real, cyclic, irreversible 2-reservoir process of a Schottky system is running between these two reservoirs exchanging the heat flows ${\overset{{}_{{}^{•}}}{Q}}_{H}\;\left(t\right)>0$ and ${\overset{{}_{{}^{•}}}{Q}}_{L}\;\left(t\right)<0$ with *H* and *L*, respectively. No mass and no work exchange appear between the reservoirs and the system undergoing the cyclic process. The heat flows depend on the contact temperature and of that of the reservoir

(22) $${\overset{{}_{{}^{•}}}{Q}}_{H}\;\left(t\right)\;=\;U_{H}\left(\Theta_{H},T_{H}\right),\;{\overset{{}_{{}^{•}}}{Q}}_{L}\;\left(t\right)\;=\;U_{L}\left(\Theta_{L},T_{L}\right),$$

representing constitutive heat conduction properties which are not specialized here, because constitutive properties are out of scope in this section. Consequently, also the material which performs the considered cyclic process is not specified: the theoretical concept of cyclic 2-reservoir processes, developed here, includes arbitrary cyclic processes of arbitrary materials. Because the non-negative definiteness of the entropy production is presupposed in the sequel, items concerning Stochastic Thermodynamics are out of scope.

The *net heat exchanges per cycle* of the cycle time τ>0 are ([$Q_{H}$]=Nm/cycle, [τ]=s/cycle)

(23) $$Q_{H}\;:=\;\int_{0}^{\tau }{\overset{{}_{{}^{•}}}{Q}}_{H}\;\left(t\right)dt\;=\;\oint {\overset{{}_{{}^{•}}}{Q}}_{H}dt,\;Q_{L}\;:=\;\int_{0}^{\tau }{\overset{{}_{{}^{•}}}{Q}}_{L}\;\left(t\right)dt\;=\;\oint {\overset{{}_{{}^{•}}}{Q}}_{L}dt.$$

Throughout the paper we will consider *heat-to-work conversion processes*, which are characterized by a non-positive work W≤0, i.e., the system delivers work per cycle to the environment. Therefore, the *First Law* for heat-to-power processes writes

(24) $$Q_{H}+Q_{L}+W=0,\;W\;\leq \;0,\;⟹\;Q_{H}\;\geq \;-Q_{L}\;>\;0.$$

**Figure 1:**
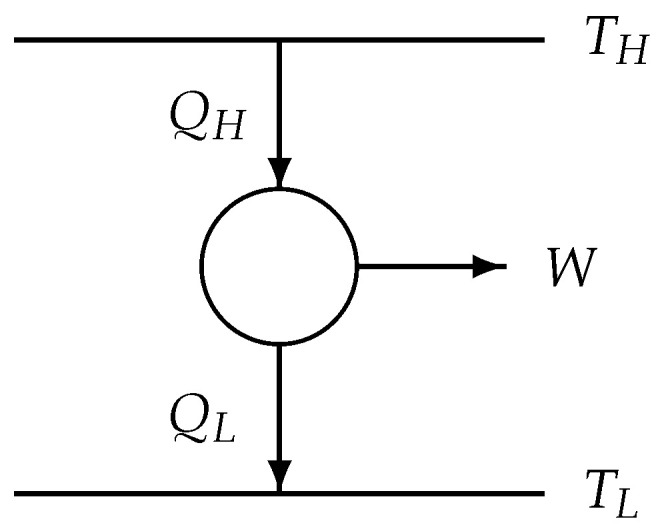
Schematic sketch of a 2-reservoir cyclic heat-to-power process. The arrows indicate the flow direction of energy (heat or work) in this particular heat-to-power process. Using the standard physics convention of heat and work entering a system being positive, one has $Q_{H}>0$, $Q_{L}<0$, and W<0.

The well-known thermodynamic diagram of a 2-reservoir cyclic heat-to-power process is shown in Figure 1.

### 4.2. Contact and Reservoir Temperatures

In order to establish a relation between the contact temperature and the reservoir temperatures below, we introduce the cycle mean values of these contact temperatures, which are defined by

(25) $$\frac{1}{\Theta^{+}}\;:=\;\frac{1}{Q_{H}}\oint \frac{{\overset{{}_{{}^{•}}}{Q}}_{H}}{\Theta_{H}}dt\;>\;0,\;\frac{1}{\Theta^{-}}\;:=\;\frac{1}{Q_{L}}\oint \frac{{\overset{{}_{{}^{•}}}{Q}}_{L}}{\Theta_{L}}dt\;>\;0.$$

The $\Theta^{+}$ and $\Theta^{-}$ are the mean values of the contact temperatures of the system generated by the cyclic process which is controlled by the constant reservoir temperatures $T_{H}$ and $T_{L}$. Starting with (14)${}_{1}$, we obtain two inequalities valid for the reservoirs *H* and *L*

(26) $$\begin{array}{ccc} \oint \frac{{\overset{{}_{{}^{•}}}{Q}}_{H}}{\Theta_{H}}dt\;\geq \;\oint \frac{{\overset{{}_{{}^{•}}}{Q}}_{H}}{T_{H}}dt & ⟶ & \frac{1}{\Theta^{+}}Q_{H}\geq \;\frac{1}{T_{H}}Q_{H}\;⟶\;\Theta^{+}\;\leq \;T_{H}, \end{array}$$

(27) $$\begin{array}{ccc} \oint \frac{{\overset{{}_{{}^{•}}}{Q}}_{L}}{\Theta_{L}}dt\;\geq \;\oint \frac{{\overset{{}_{{}^{•}}}{Q}}_{L}}{T_{L}}dt & ⟶ & \frac{1}{\Theta^{-}}Q_{L}\geq \;\frac{1}{T_{L}}Q_{L}\;⟶\;\Theta^{-}\;\geq \;T_{L}. \end{array}$$

The contact temperatures $\Theta^{+}$ and $\Theta^{-}$ belong in contrast to the reservoir temperatures $T_{H}$ and $T_{L}$ to the irreversibly running system. Because $\Theta^{+}$ and $\Theta^{-}$ are “closer to the system” than $T_{H}$ and $T_{L}$, results are expected which are more realistic than those obtained by using the reservoir temperatures.

### 4.3. Entropy Production and Efficiency

According to (13), the *entropy production per cycle* Ω appearing in a closed ($\overset{{}_{{}^{•}}}{n}{}^{e}\equiv 0$) 2-reservoir system of controlling reservoirs *H* and *L* of constant thermostatic temperatures is by use of (13)${}_{1}$ and (14)${}_{1}$ ([Ω]=Nm/(K cycle))

(28) $$0\;\leq \;\Omega \;:=\;\oint \Sigma dt\;=\;-\oint \frac{1}{\Theta }\overset{{}_{{}^{•}}}{Q}dt\;\leq \;-\oint \frac{1}{T^{*}}\overset{{}_{{}^{•}}}{Q}dt\;=\;-\frac{Q_{H}}{T_{H}}-\frac{Q_{L}}{T_{L}}.$$

This inequality represents the special form of *Clausius’ inequality* for 2-reservoir systems [56]. Using (28)${}_{3}$, the entropy production per cycle becomes by use of (25)

(29) $$0\;\leq \;\Omega \;=\;-\oint \frac{1}{\Theta }\overset{{}_{{}^{•}}}{Q}dt\;=\;-\frac{1}{\Theta^{+}}\oint {\overset{{}_{{}^{•}}}{Q}}_{H}dt-\frac{1}{\Theta^{-}}\oint {\overset{{}_{{}^{•}}}{Q}}_{L}dt\;=\;-\frac{Q_{H}}{\Theta^{+}}-\frac{Q_{L}}{\Theta^{-}}.$$

The second equality is due to the mean value theorem establishing the mean values of the system’s contact temperatures averaged over the cyclic process as already done in (26) and (27). In contrast to Clausius’ inequality (28) which represents an estimation of the entropy production, (29) is an equation for it.

From (29)${}_{4}$ it follows by taking (24) into account that

(30) $$-\frac{Q_{L}}{\Theta^{-}}\;\geq \;\frac{Q_{H}}{\Theta^{+}}\;⟶\;\frac{Q_{H}+W}{\Theta^{-}}\;\geq \;\frac{Q_{H}}{\Theta^{+}}\;⟶\;Q_{H}\left(\frac{1}{\Theta^{-}}-\frac{1}{\Theta^{+}}\right)\;\geq \;\frac{-W}{\Theta^{-}}\;\geq \;0,$$

that together with (26) and (27) results in

(31) $$T_{L}\;\leq \;\Theta^{-}\;\leq \;\Theta^{+}\;\leq \;T_{H}.$$

The *efficiency* of the 2-reservoir process is defined by the *work per cycle* and the heat input [57]

(32) $$0\;\leq \;\eta \;:=\;\frac{-W}{Q_{H}}\;=\;\frac{Q_{H}+Q_{L}}{Q_{H}}\;=\;1+\frac{Q_{L}}{Q_{H}}\;\leq \;1-\frac{\Theta^{-}}{\Theta^{+}}\;\leq \;1-\frac{T_{L}}{T_{H}}$$

and is transformed by taking (24)${}_{1}$, (30)${}_{1}$ and (31) into account. Consequently, two upper limits of the efficiency are obtained, one formulated with the contact temperatures, the other one with the reservoir temperatures.

### 4.4. Heat Exchange Coefficient, Non-Reversibility, and Power

From (32)${}_{5,6}$ follows the *heat exchange coefficient* α

(33) $$1\;\leq \;\alpha \;:=\;-\frac{Q_{H}}{Q_{L}}\;\leq \;\frac{\Theta^{+}}{\Theta^{-}}\;\leq \;\frac{T_{H}}{T_{L}}\;⟶\;Q_{H}\;=\;-\alpha Q_{L}.$$

**Figure 2:**
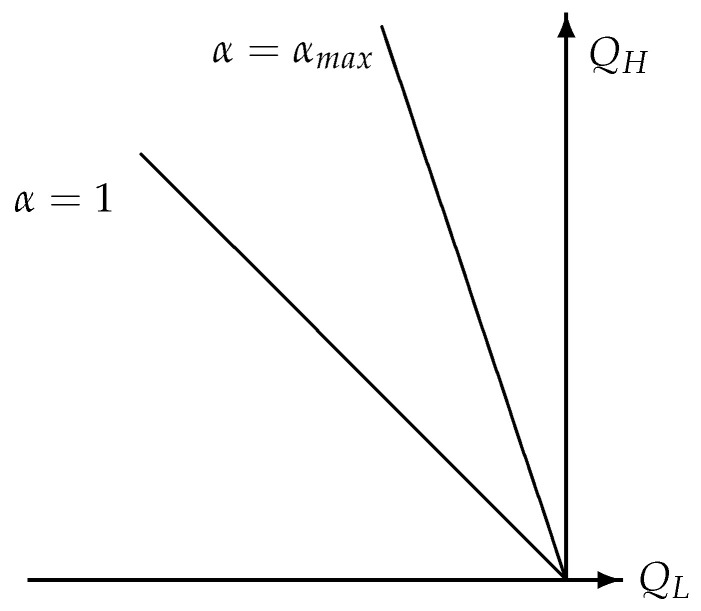
Different values of the heat exchanging coefficient α characterizing different work producing thermodynamic cyclic processes which all are located between α=1 and $\alpha =\alpha_{max}>1$.

These inequalities demonstrate that each work producing thermodynamic cyclic process belongs to a heat exchanging coefficient which is located in the angle between α=1 and $\alpha =\alpha_{max}$ in Figure 2.

By taking (33)${}_{2}$ into account, the efficiency (32)${}_{4}$ results in

(34) $$0\;\leq \;\eta \left(\alpha \right)\;=1-\frac{1}{\alpha }\;\leq \;1\;-\;\frac{1}{\alpha_{max}}\;=:\;\eta_{max}.$$

The entropy production per cycle (29)${}_{4}$ becomes by taking (28)${}_{4}$ and (33)${}_{2}$ into account

(35) $$0\leq \Omega =-\frac{Q_{H}}{\Theta^{+}}-\frac{Q_{L}}{\Theta^{-}}\;=:\;-\Lambda \left(\frac{Q_{H}}{T_{H}}+\frac{Q_{L}}{T_{L}}\right)\;=\;-\Lambda \frac{Q_{H}}{T_{L}}\left(\frac{T_{L}}{T_{H}}-\frac{1}{\alpha }\right),\;0<\Lambda \leq 1.$$

The parameter Λ is called the *non-reversibility*. Its range is generated by the inequality (28)${}_{4}$. It is defined by (35)${}_{3}$ and makes possible to replace the mean process values of the contact temperature, $\Theta^{+}$ and $\Theta^{-}$, (quantities which are difficult to determine experimentally) by one parameter $\Lambda (\alpha ,\Theta^{+},\Theta^{-},T_{H},T_{L})$ according to (35) which is limited by (35)${}_{5}$ and which describes the correction, if the contact temperatures are replaced by the reservoir temperatures. As shown Appendix A.1, the non-reversibility is

(36) $$\Lambda \left(\alpha ,\Theta^{+},\Theta^{-},T_{H},T_{L}\right)\;=\;\frac{\left(\alpha \Theta^{-}-\Theta^{+}\right)T_{H}T_{L}}{\left(\alpha T_{L}-T_{H}\right)\Theta^{+}\Theta^{-}}.$$

From (35) follows by use of (34)${}_{2}$ and (32)${}_{2}$

(37) $$T_{L}\frac{\Omega }{\Lambda }=-Q_{H}\left(\frac{T_{L}}{T_{H}}-1+1-\frac{1}{\alpha }\right)\;=\;Q_{H}\left(1-\frac{T_{L}}{T_{H}}\right)-Q_{H}\eta \;=\;Q_{H}\left(1-\frac{T_{L}}{T_{H}}\right)+W.$$

Here, Ω/Λ≥Ω is the *reservoir-related entropy production* (28)${}_{5}$, which exceeds the regular entropy production (29)${}_{4}$. Consequently, the work per cycle which is done on the system’s environment is according to (37),

(38) $$0\;\leq \;-W\;=\;Q_{H}\left(1-\frac{T_{L}}{T_{H}}\right)-T_{L}\frac{\Omega }{\Lambda }\;\leq \;Q_{H}\left(1-\frac{T_{L}}{T_{H}}\right)-T_{L}\Omega .$$

The somewhat strange fact that two different entropy productions occur (namely, Ω and Ω/Λ) is due to the side by side use of contact and reservoir temperatures, whereas the entropy production is based on the time rate of entropy necessarily formulated with the contact temperature, the work belongs to the greater reservoir-related entropy production according to (38). Another shape of (35)${}_{3,2}$ or (38)${}_{1}$ is

(39) $$\frac{Q_{H}}{T_{H}}+\frac{Q_{L}}{T_{L}}\;=\;-\frac{\Omega }{\Lambda }\;\leq \;0,\;\frac{Q_{H}}{\Theta^{+}}+\frac{Q_{L}}{\Theta^{-}}\;=\;-\Omega \;\leq \;0.$$

The *power per cycle* becomes by use of the cycle time τ and of (32)${}_{1}$

(40) $$P\;:=\;\frac{-W}{\tau }\;=\;Q_{H}\frac{\eta }{\tau }\;\geq \;0.$$

Inserting (34)${}_{2}$ and (33)${}_{6}$, the power per cycle results according to (34)${}_{2}$ in

(41) $$P\;=\;\frac{Q_{H}}{\tau }\left(1-\frac{1}{\alpha }\right)\;=\;\frac{Q_{L}}{\tau }\left(1-\alpha \right)\;=\;\frac{Q_{L}}{\tau }\frac{\eta }{\eta -1}.$$

From (38)${}_{2}$ and (40)${}_{2}$ follows

(42) $$P\;=\;\frac{P}{\eta }\left(1-\frac{T_{L}}{T_{H}}\right)-\frac{T_{L}}{\tau }\frac{\Omega }{\Lambda }\;⟹\;\left(\frac{1}{\eta }\left(1-\frac{T_{L}}{T_{H}}\right)-1\right)P\;=\;\frac{T_{L}}{\tau }\frac{\Omega }{\Lambda },$$

resulting in

(43) $$\left(1-\frac{T_{L}}{T_{H}}-\eta \right)P\;=\;\frac{T_{L}}{\tau }\frac{\Omega }{\Lambda }\eta \;⟹\;\eta \;\leq \;1-\frac{T_{L}}{T_{H}},$$

a relation which come again into consideration, if reversible processes are taken into account (53).

### 4.5. Reversible “Processes”

Because endoreversible models are considered in the sequel, reversible processes have to be defined, and the thermodynamic relations of Section 4.1, Section 4.2, Section 4.3 and Section 4.4 are translated for reversible processes. These “processes” are defined by vanishing entropy production

(44) $$\Omega_{rev}\;\equiv \;0.$$

From (39) and (44)${}_{1}$ follows *Clausius’ equality*

(45) $$\frac{Q_{H}^{rev}}{T_{H}}+\frac{Q_{L}^{rev}}{T_{L}}\;=\;0\;=\;\frac{Q_{H}^{rev}}{\Theta_{rev}^{+}}+\frac{Q_{L}^{rev}}{\Theta_{rev}^{-}},$$

by use of (31) and (24)${}_{4}$ resulting in

(46) $$0\;\geq \;Q_{H}^{rev}\left(\frac{1}{T_{H}}-\frac{1}{\Theta_{rev}^{+}}\right)\;=\;Q_{L}^{rev}\left(\frac{1}{\Theta_{rev}^{-}}-\frac{1}{T_{L}}\right)\;\geq \;0.$$

Consequently,

(47) $$\Theta_{rev}^{+}\;=\;T_{H},\;\Theta_{rev}^{-}\;=\;T_{L}$$

follows, that means, the difference between contact and reservoir temperatures vanishes for reversible processes, and from (36) and (47) follows for the non-reversibility

(48) $$\Lambda_{rev}\;=\;1.$$

Starting with (32)${}_{2}$ written down for reversible processes, (38)${}_{2}$ results by use of (34)${}_{2}$ in

(49) $$Q_{H}^{rev}\eta_{rev}\;=\;-W^{rev}\;=\;Q_{H}^{rev}\left(1-\frac{T_{L}}{T_{H}}\right)\;⟹\;\eta_{rev}=1-\frac{T_{L}}{T_{H}}\;⟹\;\alpha_{rev}=\frac{T_{H}}{T_{L}}.$$

Taking (49)${}_{3}$ into account, (38) becomes

(50) $$0\;\leq \;-W\;=\;Q_{H}\eta_{rev}-T_{L}\frac{\Omega }{\Lambda }\;\leq \;Q_{H}\eta_{rev}-T_{L}\Omega ,$$

that results in two statetments: (i) the reversible work is maximal

(51) $$\Omega \;≐\;0\;⟹\;-W_{max}\;=\;Q_{H}\left(1-\frac{T_{L}}{T_{H}}\right)\;=\;-W^{rev}$$

and (ii) the reservoir-related entropy production is maximal, if the work vanishes

(52) $$W\;≐\;0\;⟹\;{\left(\frac{\Omega }{\Lambda }\right)}_{max}\;=\;Q_{H}\left(\frac{1}{T_{L}}-\frac{1}{T_{H}}\right).$$

and finally, the power (43) becomes

(53) $$\left(\eta_{rev}-\eta \right)P\;=\;\frac{T_{L}}{\tau }\frac{\Omega }{\Lambda }\eta \;\geq \;0\;⟹\;\eta \;\leq \;\eta_{max}\;=\;\eta_{rev}.$$

The expressions for the entropy production, power and efficiency, which are derived here, will be needed in the next sections for simulating real cyclic 2-reservoir processes by endoreversible models.

### 4.6. Maximal Power and Cycle Time: The Machine Diagrams

Considering a special engine, its “fuel consumption” $Q_{H}$ and its “heat loss” $Q_{L}$ depend on the cycle time [58]

(54) $$Q_{H}\;=\;\Psi \left(\zeta \right),\;Q_{L}\;=\;\Xi \left(\zeta \right),\;\zeta \;\equiv \;\left(T_{H},T_{L},\tau \right)$$

relations which are called *machine diagrams* and which characterize the considered engine. Consequently, the machine diagrams tranfer the cycle time to the thermodynamic quantities which are discussed in Section 4

(55) $$\begin{array}{ccc} \alpha \left(\zeta \right)\;=\;-\frac{\Psi (\zeta )}{\Xi (\zeta )},\;\eta \left(\zeta \right)\;=\;1+\frac{\Xi (\zeta )}{\Psi (\zeta )},\;P\left(\zeta \right) & = & \frac{1}{\tau }\left(\Psi (\zeta )+\Xi (\zeta )\right), \end{array}$$

(56) $$\begin{array}{ccc} \Omega \left(\zeta \right)\;=\;-\Lambda \left(\vartheta \right)\left(\frac{\Psi (\zeta )}{T_{H}}+\frac{\Xi (\zeta )}{T_{L}}\right),\;\Lambda \left(\vartheta \right) & = & \chi (\zeta ,\Theta^{+},\Theta^{-}). \end{array}$$

Taking a reversible process into account, (54) becomes

(57) $$Q_{H}^{rev}\;=\;\Psi \left(\zeta_{rev}\right),\;Q_{L}^{rev}\;=\;\Xi \left(\zeta_{rev}\right),\;\zeta_{rev}\;\equiv \;\left(T_{H},T_{L},\infty \right)$$

Taking the machine diagrams (54) into consideration, the relation (55)${}_{2}$ of the efficiency depends on the cycle time. If the solubility of (55)${}_{2}$ for the cycle time is presupposed,

(58) $$\tau \;=\;\Pi (T_{H},T_{L},\eta ),$$

the power (40)${}_{2}$ results in

(59) $$0\;\leq \;\frac{P}{Q_{H}}\;=\;\frac{\eta }{\tau (\eta )},\;⟹\;\left(\frac{P}{Q_{H}}\right){|}_{\eta =0}\;=\;0,\;\left(\frac{P}{Q_{H}}\right){|}_{\eta =\eta_{rev}}\;=\;0$$

Now the question arises: Is there any efficiency $\eta^{*}$ for which the power per fuel consumption is maximal? According to (59), the answer depends on the cycle time τ(η):

(60) $$\frac{d}{d\eta }\left(\frac{P}{Q_{H}}\right)\;=\;\frac{\tau (\eta )-\eta (d\tau /d\eta )}{\tau^{2}\left(\eta \right)}\;≐\;0.$$

There is a local maximum of $P/Q_{H}$ with respect to the efficiency, because the equation

(61) $$\eta^{*}\left(\frac{d\tau }{d\eta }\right){|}_{\eta^{*}}\;=\;\tau \left(\eta^{*}\right),\;⟹\;\left(\frac{d\ln\tau }{d\eta }\right){|}_{\eta^{*}}\;=\;\frac{1}{\eta^{*}}$$

has a solution $\eta^{*}$ due to $P/Q_{H}\equiv \;|\;0$ and (59)${}_{3,4}$.

The local maximum of another quantity, P/(Ω/Λ), with respect to the efficiency is found out in the Appendix A.2. These two examples demonstrate that the machine diagrams determine for what efficiency the power is maximal. According to the machine diagrams (54), the power (55)${}_{3}$ depends on the cycle time. That is the reason why the machine diagrams have to be taken into account. If other quotients like $P/Q_{H},\;P/Q_{L}$, or P/(Ω/Λ), in which the power is measured relative to $Q_{H}$, etc., are optimized, then the maxima of these quotients belong to different efficiencies.

### 4.7. Universality

The cyclic 2-reservoir heat-to-power processes considered in the above section are universal in the following sense.

- The cyclic process between the two heat reservoirs is arbitrary: it may be a Carnot, Otto, Diesel, Brayton, or another cyclic reversible or irreversible process.
- The working material which perform this cyclic process under control of the two heat reservoirs is arbitrary: it may be a perfect or real gas, a fluid, a liquid crystal, or radiation in classical or quantumtheoretical description, the only restriction is that the chosen substance allows such a cyclic 2-reservoir process.

Consequently, the general concepts developed above in Section 4 can be applied to the items considered below. The simulation of an irreversible cyclic 2-reservoir heat-to-power process by different endoreversible models which do not represent real processes because of their reversible parts. To explain what simulation means, two well-known examples are again considered for remembrance [58]: the reversible Carnot process with heat leak in Section 6.1 and the Curzon–Ahlborn model in Section 6.2.

## 5. Simulation and Modeling

### 5.1. Process Class

All real cyclic 2-reservoir processes can be described by the reservoir temperatures, by the cycle mean values of the contact temperatures (25), by the heat exchanges (23), and by the cycle time. Instead of the contact temperatures, one can use for our purpose the non-reversibility Λ. Consequently, a 6-dimensional manifold, the *process class* is introduced:

(62) $$z\;:=\;\left(T_{H},T_{L},\Lambda ,Q_{H},Q_{L},\tau \right)\;\in \;M^{6}.$$

The physical meaning of the parameters spanning this manifold induces some restrictions: $T_{H}>T_{L}>0,\;\tau >0,\;Q_{H}\geq -Q_{L}>0$ satisfying (33)${}_{2}$, 0<Λ≤1, according to (35)${}_{5}$, depending on the reservoir and contact temperatures and on the heat exchanges according to (36).

For arbitrary, but fixed allowed values of the quantities $\left(T_{H},T_{L},\Lambda ,Q_{H},Q_{L},\tau \right)$, we call *z* a *process class* and $M^{6}$ the *set of all process classes*. According to its definition, the process class contains all processes having the same values for *z*, not implying that the process mean values of the contact temperatures are equal: Consider two processes, *I* and II, of the same process class

(63) $$\Lambda_{I}\;=\;\frac{\left(\alpha \Theta_{I}^{-}-\Theta_{I}^{+}\right)T_{H}T_{L}}{\left(\alpha T_{L}-T_{H}\right)\Theta_{I}^{+}\Theta_{I}^{-}}\;=\;\Lambda_{II}\;=\;\frac{\left(\alpha \Theta_{II}^{-}-\Theta_{II}^{+}\right)T_{H}T_{L}}{\left(\alpha T_{L}-T_{H}\right)\Theta_{II}^{+}\Theta_{II}^{-}},$$

resulting in

(64) $$\frac{\alpha \Theta_{I}^{-}-\Theta_{I}^{+}}{\Theta_{I}^{+}\Theta_{I}^{-}}\;=\;\frac{\alpha \Theta_{II}^{-}-\Theta_{II}^{+}}{\Theta_{II}^{+}\Theta_{II}^{-}}\;⟶\;\Theta_{I}^{\pm }\;\mathrm{may}\;\mathrm{be}\;\mathrm{different}\;\mathrm{from}\;\Theta_{II}^{\pm }.$$

Introducing the process class, we concern ourselves no longer with the particular time dependence of the heat flows during the cyclic process, but we group together all processes having the same values of *z* forming the process class. All processes of a process class are equivalent to each other.

### 5.2. Simulating Processes

Now, we want to simulate a real irreversible cyclic 2-reservoir heat-to-power process which is contained in the process class (62). That means, we have to replace the original irreversible cyclic process by a special other one. Of course this replacement is not unique: there are many other processes simulating the original one. “Simulating” means that the process replacing the original one has the same *z* as the original process: Simulating processes ${}_{SP}$ and original process ${}_{OP}$ belong to the same process class *z* [58]

(65) $$z_{OP}\;=\;z_{SP}^{I}\;=\;z_{SP}^{II}\;=\;z_{SP}^{III}\;=\;\ldots \ldots$$

Whereas the original process is a real running one, a simulating process may also be a real running one, but also reversible “processes” (not real running) are allowed. Because the simulating process is in the same process class (62) as the original one, it cannot be distinguished from the original process by elements of *z*. These simulating processes can be modeled differently. Here, we are using endoreversible models, but other modeling for generating simulating processes is possible.

### 5.3. Process Family and Machine Diagrams

The heat exchanges $Q_{H}$ and $Q_{L}$ of a real running cyclic engine between the fixed controlling heat reservoirs of the temperatures $T_{H}$ and $T_{L}$ depend on the cycle time τ. Consequently, the *process family* of such an engine is described by a family of subsets of the process class

(66) $$Z\left(\tau \right)\;:=\;(T_{H},T_{L},\Lambda \left(\tau \right),Q_{H}\left(\tau \right),Q_{L}\left(\tau \right),\tau )$$

with the cycle time as a family parameter. The Z(τ) which characterize the engine are denoted as *machine diagrams*. These machine diagrams group together process classes by making its variables dependent of each other. The process class depicts the variables of a 2-reservoir system, whereas the process family describes the constitutive properties of the considered engine.

### 5.4. Endoreversible Models

For simulating processes of an engine, endoreversible models are here used because they can be of nearly arbitrary complexity [25,26]. In this paper, a reversible Carnot “process” combined with an irreversible transport process like the Fourier or the Newton heat conduction, which simulate the entropy production of the original process, is chosen as an endoreversible model. Consequently, two steps appear in the simulation procedure: the reversibility condition related to the Carnot process and irreversibility conditions related to the entropy production. These two steps will be reflected in two corresponding types of *simulation parameters:* The first and second simulation parameters.

In the next section, we will consider endoreversible systems, and we will show how to construct special simulation parameters which determine the simulating process.

## 6. Simulation by Special Endoreversible Models

Explaining the concept of simulation [58] in more detail, two well-known endoreversible models are chosen: the reversible Carnot process with a Fourier heat leak and the Curzon–Ahlborn model with Newton heat conduction. We make this choice because these two models have accompanied the historical development of Finite-Time Thermodynamics (FTT): they are chosen because everyone is familiar with them, helping to understand what simulation means.

### 6.1. Reversible Carnot “Process” with Fourier Heat Leak

**Figure 3:**
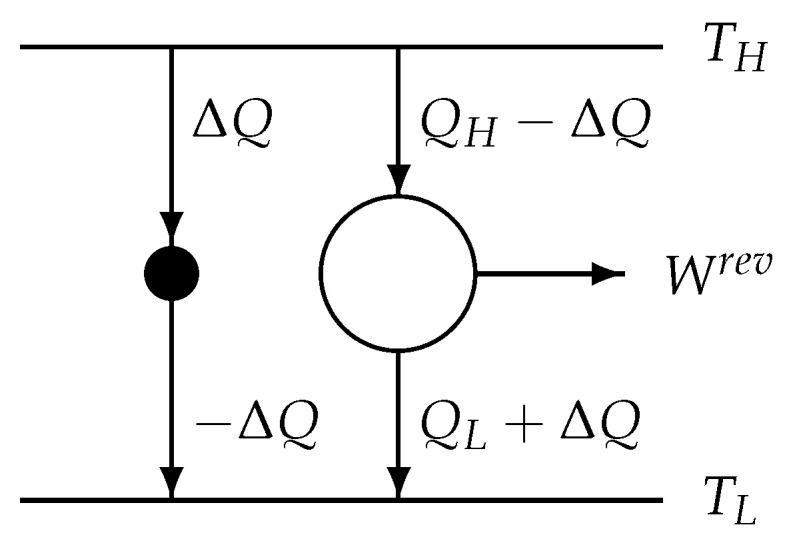
Model structure of the reversible Carnot engine with heat leak ΔQ. The black dot symbolizes that part of the endoreversible model through which the heat leak flows. From the perspective of the endoreversible model (black dot + white circle), the same total heat exchanges ($\Delta Q+Q_{H}-\Delta Q$ and $-\Delta Q+Q_{L}+\Delta Q$) as in the simulated process class occur: $Q_{H}$ and $Q_{L}$.

The model structure of the reversible Carnot process with heat leak [59] is shown in Figure 3. The reservoir-related entropy production per cycle (28)${}_{5}$ of the original process can be identically transformed into

(67) $$\frac{\Omega }{\Lambda }\;=\;-\frac{Q_{H}-\Delta Q}{T_{H}}\;-\;\frac{Q_{L}+\Delta Q}{T_{L}}\;+\;\Delta Q\left(\frac{1}{T_{L}}-\frac{1}{T_{H}}\right).$$

Because the heat leak can only be described by taking place between the heat reservoirs, the expression (35)${}_{3}$ which contains the reservoir temperatures and the non-reversibility is chosen instead of (35)${}_{2}$ which is defined by using the mean values of the contact temperatures. The introduced *heat leak per cycle* ΔQ is according to (67) arbitrary without influencing the process class *z* (62).

For constructing a particular endoreversible model, we choose ΔQ in such a way, that the sum of the first two terms on the right-hand side of (67) become zero, thus representing a Clausius’ equality describing a reversible process

(68) $$-\frac{Q_{H}-\Delta Q}{T_{H}}\;-\;\frac{Q_{L}+\Delta Q}{T_{L}}\;≐\;0.$$

This *reversibility condition* represents a reversible process having the heat exchanges $Q_{H}-\Delta Q$ and $Q_{L}+\Delta Q$ between the system and the two controlling reservoirs of the temperatures $T_{H}$ and $T_{L}$, respectively (see Figure 3). The reversible work

(69) $$-W^{rev}\;=\;\left(Q_{H}-\Delta Q\right)+\left(Q_{L}+\Delta Q\right)\;=\;-W$$

is equal to that of the original process. According to (68), the reservoir-related entropy production per cycle (67) of the original process results in

(70) $$\frac{\Omega }{\Lambda }\;=\;\Delta Q\left(\frac{1}{T_{L}}-\frac{1}{T_{H}}\right)\;\geq \;0\;⟹\;\Delta Q\geq 0.$$

The reversibility condition (68) determines the heat leak ΔQ which is connected to the entropy production. From (70) follows with (49)${}_{3}$

(71) $$\frac{\Omega }{\Lambda }\frac{T_{H}T_{L}}{T_{H}\left(1-T_{L}/T_{H}\right)}\;=\;\Delta Q\;=\;\frac{\Omega T_{L}}{\Lambda \eta_{rev}}.$$

The heat leak ΔQ is called a *first simulation parameter*. The endoreversible model of a real 2-reservoir process class is determined by specializing this first simulation parameter which is given by the reversibility condition (68) resulting in (71).

Now a *second simulation parameter* $\lambda^{hl}$ is introduced by a “constitutive equation” for the heat leak per cycle

(72) $$\Delta Q\;=:\;\lambda^{hl}\tau \left(\frac{1}{T_{L}}-\frac{1}{T_{H}}\right)\;=\;\lambda^{hl}\tau \frac{\eta_{rev}}{T_{L}}\;⟹\;\lambda^{hl}\geq 0.$$

This equation looks like a Fourier heat conduction ansatz, but it is not, because (72) determines the “heat conductivity” $\lambda^{hl}$ which of course is in general not a constant, but a function of $Q_{L},\alpha$, and τ according to (72), (71) and (56)${}_{1}$. From (72) follows with (71)

(73) $$\lambda^{hl}\left(\zeta \right)\;=\;\frac{1}{\tau }\frac{\Omega (\zeta )}{\Lambda }{\left(\frac{T_{L}}{\eta_{rev}}\right)}^{2},$$

and (72)${}_{1}$ inserted into (70) results in

(74) $$\frac{\Omega (\zeta )}{\Lambda }\;=\;\lambda^{hl}\left(\zeta \right)\tau {\left(\frac{1}{T_{L}}-\frac{1}{T_{H}}\right)}^{2}\;\geq \;0,\;\zeta \;=\;\left(T_{H},T_{L},\tau \right)$$

according to (54). The equations (73) and (74) demonstrate that the cycle time dependence of the “heat conductivity” induced by the machine diagrams has to be taken into account.

For given temperatures of the heat reservoirs, the simulation parameter $\lambda^{hl}$ depends via the reservoir-related entropy production per cycle on the cycle time. If $\lambda^{hl}$ would be set constant,

(75) $$\frac{\Omega }{\tau \Lambda }\;≐\;const.\;⟹\;-\frac{1}{\tau }\left(\frac{\Psi (\zeta )}{T_{H}}+\frac{\Xi (\zeta )}{T_{L}}\right)\;=\;const.$$

follows according to (73) and (56). However, then for arbitrary machine diagrams, (75)${}_{2}$ is in general not satisfied because its LHS depends on the cycle time. Only very special machine diagrams would make the LHS constant. Consequently, the reversible Carnot process with Fourier heat leak and an as constant chosen “heat conduction coefficient” $\lambda^{hl}$ does not represent a simulation of a general real running irreversible engine and is thus not suited for a general simulation task.

In the reversible case we obtain according to (44)${}_{1}$ from (71), that there is no heat leak

(76) $$\Delta Q_{rev}\;=\;0.$$

From (73) it follows that

(77) $$\lambda^{hl}\tau {\left(\frac{\eta_{rev}}{T_{L}}\right)}^{2}\;=\;\frac{\Omega }{\Lambda }\;=\;-\frac{Q_{H}}{T_{H}}-\frac{Q_{L}}{T_{L}}.$$

As shown in the Appendix A.3, we obtain from (77) the power

(78) $$P\;=\;\lambda^{hl}\frac{1}{T_{H}}\frac{{\left(\alpha_{rev}-1\right)}^{2}}{\alpha_{rev}-\alpha }\left(\alpha -1\right).$$

In summary, the original 2-reservoir process is simulated by an endoreversible model consisting of the reversible part described by (68) (the right-hand part in Figure 3), and of an irreversible heat conducting part, the heat leak (the left-hand part in Figure 3), described by (71)${}_{2}$. The endoreversible model undergoes the same “process” as the original one: the original process is simulated by an endoreversible model.

Another example of endoreversible modeling is considered in the next section.

### 6.2. Curzon–Ahlborn Model

**Figure 4:**
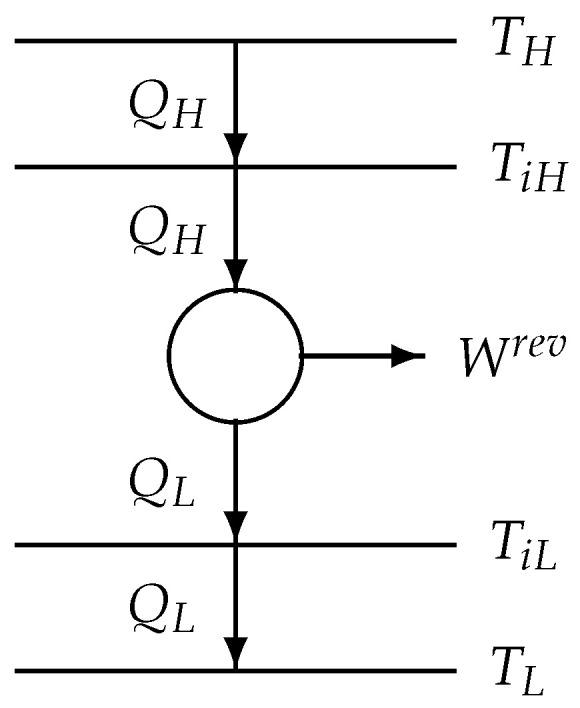
Model structure of the Curzon–Ahlborn model.

Now the original 2-reservoir process class (62), shown in Figure 1, is simulated by using another endoreversible model, the Curzon–Ahlborn model [1] with two internal temperatures $T_{iH}$ and $T_{iL}$, $T_{H}>T_{iH}>T_{iL}>T_{L}$ (see Figure 4). Because the situation is as in Figure 1 represented, we can use the results of Section 4.

The entropy production (35)${}_{3}$ is now identically transformed into

(79) $$\Omega \;=\;\Lambda \left(-\frac{Q_{H}}{T_{H}}-\frac{Q_{L}}{T_{L}}\right)=-\Lambda \left(\frac{Q_{H}}{T_{iH}}+\frac{Q_{L}}{T_{iL}}\right)+\Lambda Q_{H}\left(\frac{1}{T_{iH}}-\frac{1}{T_{H}}\right)+\Lambda Q_{L}\left(\frac{1}{T_{iL}}-\frac{1}{T_{L}}\right),$$

by introducing $T_{iH}$ and $T_{iL}$ as two *first simulation parameters*. The reversibility condition of the reversible part of the endoreversible Novikov process is chosen as

(80) $$\frac{Q_{H}}{T_{iH}}+\frac{Q_{L}}{T_{iL}}\;≐\;0\;⟹\;\alpha \;=\;\frac{T_{iH}}{T_{iL}},\;\eta \;=\;1-\frac{T_{iL}}{T_{iH}},$$

that means, one of the first simulation parameters can be freely chosen. From (79) it follows that by use of (41)${}_{2}$, the reservoir-related entropy production is

(81) $$\frac{\Omega }{\Lambda }\;=\;Q_{H}\left(\frac{1}{T_{iH}}-\frac{1}{T_{H}}\right)+Q_{L}\left(\frac{1}{T_{iL}}-\frac{1}{T_{L}}\right)\;=\;\frac{Q_{L}}{T_{H}}\left(\alpha -\alpha_{rev}\right)\;=\;\frac{-W}{T_{H}}\frac{\left(\alpha -\alpha_{rev}\right)}{1-\alpha }.$$

It is evident that the reservoir-related entropy production does not depend on the two first simulation parameters because α and *W* are determined by the original process according to (33)${}_{2}$ and (24)${}_{1}$.

Two *second simulation parameters* $\lambda_{H}$ and $\lambda_{L}$ are introduced generating “constitutive equations” in the same fashion as in (72) which represent definitions of $\lambda_{H}$ and $\lambda_{L}$

(82) $$Q_{H}\;=:\;\lambda_{H}\tau \left(T_{H}-T_{iH}\right),\;Q_{L}\;=:\;\lambda_{L}\tau \left(T_{L}-T_{iL}\right),\;\lambda_{H},\lambda_{L}>0,$$

and the reservoir-related entropy production (81)${}_{2}$ results in

(83) $$\frac{\Omega }{\Lambda }\;=\;\frac{\lambda_{L}}{T_{H}}\tau \left(T_{L}-T_{iL}\right)\left(\alpha -\alpha_{rev}\right).$$

Because the LHS of (83) is independent of $T_{iL}$ according to (81)${}_{3}$, $\lambda_{L}$ depends on $T_{iL}$. A simple (and boring) calculation in the Appendix A.4 (A17) results in

(84) $$\frac{\Omega }{\Lambda }\;=\;\tau \frac{\lambda_{L}\lambda_{H}}{\lambda_{L}+\lambda_{H}}\left(\frac{\alpha_{rev}}{\alpha }-1\right)\left(1-\frac{\alpha }{\alpha_{rev}}\right).$$

Starting with (32)${}_{2}$ and (82)${}_{1}$, a simple, but well known [58] (also boring) calculation presented in Appendix A.5 (A21) results in

(85) $$P\;=\;\lambda_{H}\left(T_{H}-T_{iH}\right)\eta \;=\;\frac{\lambda_{L}\lambda_{H}}{\lambda_{L}+\lambda_{H}}T_{H}\frac{\eta_{rev}-\eta }{1-\eta }\eta .$$

This expression is of course different from (78) due to the different models for the original process, although the values of the power in (78) and (85) are equal to that of the original process of the engine in (40)${}_{2}$.

From (82) it follows that

(86) $$\lambda_{H}\tau \;=\;\frac{Q_{H}}{T_{H}-T_{iH}},\;\lambda_{L}\tau \;=\;\frac{Q_{L}}{T_{L}-T_{iL}},$$

A short calculation results in

(87) $$\begin{array}{ccc} \frac{\lambda_{L}\lambda_{H}}{\lambda_{L}+\lambda_{H}} & = & \tau^{-1}\frac{Q_{H}Q_{L}}{Q_{L}\left(T_{H}-T_{iH}\right)+Q_{H}\left(T_{L}-T_{iL}\right)}\;=\;\tau^{-1}\frac{Q_{H}}{T_{H}-T_{iH}-\alpha \left(T_{L}-T_{iL}\right)}\;=\;\\ & = & \tau^{-1}\frac{Q_{H}}{T_{H}-\alpha T_{L}} \end{array}$$

demonstrating that in general

(88) $$\frac{\lambda_{L}\lambda_{H}}{\lambda_{L}+\lambda_{H}}\;=\;F\left(Q_{H},\alpha ,T_{H},T_{L},\tau \right)\;\neq \;const.$$

The heat exchange coefficient (33)${}_{2}$ becomes with (82) and (80)${}_{2}$

(89) $$-\frac{Q_{H}}{Q_{L}}\;=\;\alpha \;=\;-\frac{\lambda_{H}}{\lambda_{L}}\frac{\left(T_{H}-T_{iH}\right)}{\left(T_{L}-T_{iL}\right)}\;=\;\frac{T_{iH}}{T_{iL}},$$

resulting in

(90) $$\lambda_{L}\;=\;\frac{T_{iL}\left(T_{H}-T_{iH}\right)}{T_{iH}\left(T_{iL}-T_{L}\right)}\lambda_{H}\;=\;\frac{T_{H}-\alpha T_{iL}}{\alpha (T_{iL}-T_{L})}\lambda_{H}.$$

According to (80), one of the first simulation parameters can be chosen freely. With respect to the machine diagrams (54) and (55)${}_{1}$, $T_{iL}$ is not determined by the original process

(91) $$T_{iH}\left(\zeta \right)\;=\;\alpha \left(\zeta \right)T_{iL},\;\zeta \;\equiv \;\left(T_{H},T_{L},\tau \right),$$

and (86) results in

(92) $$\lambda_{H}\left(\zeta ;T_{iL}\right)\;=\;\frac{1}{\tau }\frac{\Psi (\zeta )}{T_{H}-\alpha \left(\zeta \right)T_{iL}},\;\lambda_{L}\left(\zeta ;T_{iL}\right)\;=\;\frac{1}{\tau }\frac{\Xi (\zeta )}{T_{L}-T_{iL}}.$$

Consequently, $\lambda_{H}$ and $\lambda_{L}$ are as $T_{iL}$ not determined by the original process of the engine. However, because F in (88) does not depend on $T_{iL}$, the reservoir-related entropy production (84) and the power (85) are determined by the original process.

Up to here, the problem was as follows. How can the original process described in Section 4 be simulated by use of an endoreversible model? In the next section, the question is inverted: Given an endoreversible model, what real running process can belong to it?

## 7. Reconstruction, Parameter and Model Diagrams

During the 45 years since the Curzon–Ahlborn paper [2], a huge number of endoreversible models have been considered. Many of these models were simple ones considering continuously running or cyclic processes and analyzed basic features like power production or efficiencies of heat-to-power conversions. Most of those can be considered as paradigmatic examples not directly connected to any real existing heat-to-power engine. Nonetheless, the question arises, whether there exists a connection between these numerous not running endoreversible models and real running processes. More precisely, is it possible to generate an endoreversible model such that it can approximate a real running engine? This question is now shortly discussed for the example of the Curzon–Ahlborn model (CAM) described in Section 6.2.

For distinguishing all quantities *X* of the endoreversible model (CAM) from those of a real running engine, they are denoted by $X^{+}$. The reconstruction procedure starts always with the choice of the endoreversible model; here, the CAM and its thermodynamic relations which are described in Section 6.2. The simulation parameters which are determined by simulation can now be chosen freely and are denoted as *reconstruction parameters* for characterizing their free choice in contrast to the simulation parameters.

In the endoreversible CAM we have two first *reconstruction parameters*, $T_{iH}^{+}$ and $T_{iL}^{+}$, and two second *reconstruction parameters*, $\lambda_{H}^{+}$ and $\lambda_{L}^{+}$. These are not independent of each other, because of the reversibility condition (80)${}_{1}$

(93) $$\frac{Q_{H}^{+}}{T_{iH}^{+}}+\frac{Q_{L}^{+}}{T_{iL}^{+}}\;=\;0$$

where, according to (82), the heat exchanges $Q_{H}^{+}$ and $Q_{L}^{+}$ depend on the second *reconstruction parameters* $\lambda_{H}^{+}$ and $\lambda_{L}^{+}$ as well as on the first *reconstruction parameters* $T_{iH}^{+}$ and $T_{iL}^{+}$.

(94) $$\begin{array}{c} Q_{H}^{+}\;=\;\lambda_{H}^{+}\tau^{+}\left(T_{H}-T_{iH}\right),\;Q_{L}^{+}\;=\;\lambda_{L}^{+}\tau^{+}\left(T_{L}-T_{iL}\right),\;\lambda_{H}^{+},\lambda_{L}^{+}>0. \end{array}$$

The first and second *reconstruction parameters* are fixed as functions of the cycle time $\tau^{+}$ through the choice of their *parameter diagrams*

(95) $$\begin{array}{cccc} T_{iH}\; & =\;T_{iH}\left(\tau^{+}\right), & \;T_{iL}\; & =\;T_{iL}\left(\tau^{+}\right), \end{array}$$

(96) $$\begin{array}{cccc} \lambda_{H}^{+}\; & =\;\lambda_{H}^{+}\left(\tau^{+}\right), & \;\lambda_{L}^{+}\; & =\;\lambda_{L}^{+}\left(\tau^{+}\right), \end{array}$$

which have to respect the usual positivity requirements for temperatures and heat conductances as well as the reversibility condition (80). With that choice also the heat exchange coefficient and internal efficiency can be determined

(97) $$\alpha^{+}\;=\;\frac{T_{iH}^{+}}{T_{iL}^{+}}\;=\;-\frac{Q_{H}^{+}}{Q_{L}^{+}},\;\eta^{+}\;=\;1-\frac{T_{iL}^{+}}{T_{iH}^{+}}.$$

as well as the power and the reservoir-related entropy production

(98) $$\begin{array}{c} P^{+}\;=\;\lambda_{H}^{+}\left(T_{H}-T_{iH}\right)\eta^{+}\;=\;\frac{\lambda_{L}^{+}\lambda_{H}^{+}}{\lambda_{L}^{+}+\lambda_{H}^{+}}T_{H}\frac{\eta_{rev}-\eta^{+}}{1-\eta^{+}}\eta^{+}, \end{array}$$

(99) $$\begin{array}{c} {\left(\frac{\Omega }{\Lambda }\right)}^{+}\;=\;\tau^{+}\frac{\lambda_{L}^{+}\lambda_{H}^{+}}{\lambda_{L}^{+}+\lambda_{H}^{+}}\left(\frac{\alpha_{rev}^{+}}{\alpha^{+}}-1\right)\left(1-\frac{\alpha^{+}}{\alpha_{rev}}\right). \end{array}$$

From here the endoreversible analog to the machine diagrams, the *model diagrams*, are generated

(100) $$Q_{H}^{+}\left(\tau^{+}\right)\;=\;\lambda_{H}^{+}\left(\tau^{+}\right)\tau^{+}\left(T_{H}-\alpha^{+}T_{iL}^{+}\left(\tau^{+}\right)\right),\;Q_{L}^{+}\left(\tau^{+}\right)\;=\;\lambda_{L}^{+}\left(\tau^{+}\right)\tau^{+}\left(T_{L}-T_{iL}^{+}\left(\tau^{+}\right)\right),$$

which can be compared with the machine diagrams of an engine.

Consequently, the quality of an endoreversible model for describing an engine can be tested by a comparison of the model diagrams with the corresponding machine diagrams. With the choice of simple parameter diagrams—like constant heat conductances—it is apparent, that the model diagrams will not model given machine diagrams exactly, but they may serve as a more or less good approximation. Then, an approximation procedure can be established: changing the reconstruction parameters and the parameter diagrams of the endoreversible model in such a way that the resulting model diagrams are approaching the given machine diagrams of the engine. If the parameter diagrams are chosen in such a way that model diagrams and machine diagrams are identical, the reconstruction annuls the simulation.


−− −− −− −− −− −− −− −− −− −− −− −− −− −− −− −− −− −− −− −− −− −− −− −− −− −− −− −− −− −− −− −− −−Simulation:engine + machine diagrams⟶            ⟶endoreversible model + simulation parameter diagrams−− −− −− −− −− −− −− −− −− −− −− −− −− −− −− −− −− −− −− −− −− −− −− −− −− −− −− −− −− −− −− −− −−Reconstruction:endoreversible model + reconstruction parameter diagrams⟶            ⟶model diagrams + model of engine−− −− −− −− −− −− −− −− −− −− −− −− −− −− −− −− −− −− −− −− −− −− −− −− −− −− −− −− −− −− −− −− −−


Simulation and reconstruction have different starting points. For simulation, the process family of an engine with its machine diagrams is given, whereas for reconstruction, the reconstruction parameter diagrams together with the structure of the considered endoreversible model are at the beginning.

Diagrams which introduce the cycle time to the thermodynamic quantities are necessary in both cases: in simulation these are the machine diagrams, whereas in reconstruction a cycle time is introduced by the parameter diagrams. It are the parameter and the resulting model diagrams which generate the utility of endoreversible models by reconstructing them to models of an engine.

## 8. Simulation, Modeling, Reconstruction, and FTT

As already pointed out in the introduction, the original goal of Finite-Time Thermodynamics was to capture the influence of “haste” on the performance of thermodynamic processes and in particular on heat-to-power conversion. The observed efficiencies differ widely from the well-known Carnot efficiency, and thus better estimates were desired. Moreover, the knowledge on realistic performance measures allows to ask whether existing processes can be optimized by minimizing the dissipation necessary to reach a target output.

If a particular heat engine performing a cyclic 2-reservoir process is considered, then by the choice of an operating point the process class (62) is fixed. To find out whether that is a good or bad operating point one needs other processes [58] with which a comparison with the original process can be performed. Such processes can be taken either from the machine diagrams of a real engine, or from the model diagrams induced by reconstruction parameter diagrams of an (endoreversible) model. While the first point of view puts its focus on the *simulation* of the process and can thus be used to optimize the particular heat engine under consideration, the second view puts its focus on the *modeling* of the process and its *reconstruction*, thus allowing for simple calculations and for insight into (more) realistic efficiencies for instance at maximum power.

The first point of view—simulating the processes of real engines by an endoreversible model—becomes particularly important, when such a model becomes part of a larger endoreversible description. Using *simulations* enlarges the tool box of Endoreversible Thermodynamics and allows to include irreversible engines with given machine diagrams into the description [60]. Using the appropriate complexity, the interesting entropy production sources can be *quantitatively* mapped into the description. Based on the resulting features, the design and optimization of the entire system can then be performed. Such building blocks have for instance been used to model a full hydraulic recuperation system for trucks [61]. In that sense Endoreversible Thermodynamics has left the level treating only simple but paradigmatic cases; it can now also be used as an engineering design tool.

The second point of view is particularly useful if one searches for paradigmatic models with which important insights can be gained. It turned out that the efficiencies calculated at maximum power for the Curzon–Ahlborn model are independent of the values of the used heat conductances. The differences between the efficiencies obtained for different types of heat conduction (Newton, Fourier etc.) immediately show that the results depend very much on the chosen model. Recently the search for such paradigmatic models have led to the study of Novikov models with fluctuating heat bath. The results again are paradigmatic in sense of a certain universality with respect to stochastic features of the fluctuations [62,63,64].

Endoreversible Thermodynamics has also shown its usefullness in describing systems in a relatively coarse fashion to analyze for instance the optimization potential for a given real process. As an example we mention the optimization of the piston motion in cyclic heat engines, which has been investigated for engines with Otto [65,66,67], Diesel [68,69,70,71], and Miller cycles [72], as well as the special paths needed for light-driven engines [73,74,75,76,77,78]. For instance, in [32] this approach has been used to get a good guess of the potential power output gains for an alpha-type Stirling engine by using an optimized control of the piston motion. Based on a special class of piston motions, the power-optimized motion showed a power output gain of about 50% and more over a large parameter range. As long as the goal is to establish whether gains of 10% as in the Diesel case [68] or 50% as in this case are possible, the simple model with fixed transport coefficients, which can be varied during the analysis, suffices thus providing a fast and effective solution approach.

Finally, if the performance of a real heat-to-power thermodynamic device should be analyzed, simple endoreversible heat engine models with one or two fixed model parameters will not capture the important features due to their oversimplified structure. In order to reach the level of an engineering description, the complexity of the *model structure* can be increased. It is one of the great advantages of endoreversible modeling, that it allows to adjust the level of complexity to the desired level of accuracy by providing more reconstruction parameters. After having introduced the endoreversibility condition, and following the philosophy of FFT, the most important dissipative loss terms of the engine are modeled and then supplemented by model elements for further dissipative loss terms of less and less importance until the appropriate modeling level is reached. Together with its reconstruction parameter diagrams, the endoreversible model can then be checked against experimental data.

## 9. Glossary

**engine** means always a real running engine with its → machine diagrams.

**process** without addendum means a real running irreversible process. The other “processes” need an addendum: → reversible or → endoreversible.

**reversible** means vanishing → entropy production.

**endoreversible model** is a system of → reversible parts which interact irreversibly with each other, thus generating → entropy production. → “Processes” in such a system are called endoreversible. It is characzerized by a chosen structure and parameters (→ simulation parameters or → reconstruction parameters).

**set of process classes** is the manifold $M^{6}$ spanned by the independent data of all cyclic 2-reservoir processes $\left(T_{H},T_{L},\Lambda ,Q_{H},Q_{L},\tau \right)$.

**process class**$z=(T_{H},T_{L},\Lambda ,Q_{H},Q_{L},\tau )$ is a member of the → set of all process classes $M^{6}$. It includes all → processes (reversible or irreversible, real running or endoreversible) with the same *z*.

**non-reversibility**Λ, 0<Λ≤1, is a function of $\left(T_{H},T_{L},Q_{H},Q_{L},\tau \right)$ and of the cycle mean values of the contact temperature $\left(\Theta^{+},\Theta^{-}\right)$. The non-reversibility distinguishes between different → entropy productions

**process family** is given by → machine diagrams or by → model diagrams. It includes all → process classes belonging to the same → engine or → endoreversible model.

**machine diagrams** determine $\Lambda \left(\tau \right),Q_{H}\left(\tau \right)$, and $Q_{L}\left(\tau \right)$ as functions of the cycle time τ. The sub-set $\left(T_{H},T_{L},\Lambda \left(\tau \right),Q_{H}\left(\tau \right),Q_{L}\left(\tau \right),\tau \right)$ of the → set of all process classes represents a → process family with the cycle time τ as a family parameter.

**entropy production** appears twofold: entropy production with respect to the cycle mean values of the contact temperature of the considered system, and entropy production with respect to the reservoir temperatures of the controlling heat bathes. These two kinds of entropy production are connected by the → non-reversibility.

**simulation parameters** characterize an → endoreversible model and performing a → simulation, their functional dependence on τ, → the simulation parameter diagram, is determined by the → machine diagram

**simulation** is the procedure of generating an → endoreversible model including the choice of appropriate → simulation parameter diagrams (for the → simulation parameters), such that the resulting model diagram belongs to the same → process family as a *given* → engine.
  **simulation parameter diagrams** determine the τ-dependence of the → simulation parameters.

**reconstruction** is the procedure of starting with an → endoreversible model including *chosen* → reconstruction parameter diagrams (for the → reconstruction parameters) and then generating a → model diagram. Consequently, reconstruction is the reciprocal procedure to simulation.

**reconstruction parameters** correspond to the → simulation parameters by change of name. The difference between them: The values of the → simulation parameters are determined by the → simulation parameter diagrams, which in turn are determined by the → machine diagrams of the → engine, whereas the values of the reconstruction parameters can be chosen.

**model parameters** are → simulation or → reconstruction parameters.

**reconstruction parameter diagrams** determine the τ-dependence of the → reconstruction parameters. The reconstruction parameter diagrams can be chosen freely. Inserted into the thermodynamic relations of the chosen → endoreversible model, they generate the → model diagrams.

**model diagrams** are generated for a given → endoreversible model from its *chosen* → parameter diagrams. The model diagrams include the same variables as the → machine diagrams, but they differ from each other: → machine diagrams belong to an → engine, whereas parameter diagrams generated by → reconstruction belong to an → endoreversible model. Because model diagrams and → machine diagrams have the same range, they can be compared, that means, the quality of an → endoreversible model for describing an → engine can be tested by a comparison of the model diagrams with the corresponding → machine diagrams.

## 10. Summary

The paper starts with the basic facts of nonequilibrium thermodynamics of discrete systems: time rate of entropy of compound systems and cyclic processes are considered and the contact temperature is introduced enforcing the nonequilibrium entropy to be a state function and generating a Clausius-like inequality describing the 2nd law. Using these items, irreversible and reversible cyclic 2-heat-reservoir heat-to-power processes are discussed elucidating the difference beween contact and reservoir temperatures. This difference generates two different entropy productions, a contact temperature orientated and a reservoir-related one. This difference is described by a contact and reservoir temperature dependent function, called non-reversibility. The definitions of efficiency and heat exchange coefficient are not affected by the difference beween contact and reservoir temperatures, but their values differ because of different heat exchanges due to different contact and reservoir temperatures. To bring the cycle time to the thermodynamical quantities, machine diagrams have to be introduced. At the end of this section, the universality of the considered cyclic 2-reservoir heat-to-power processes is emphasized.

Then the concepts of endoreversible modeling, simulation and reconstruction are introduced:

- Endoreversible modeling means: Creating a model structure of reversible systems which interact by irreversible exchange processes supplemented with model parameters.
- Simulation means: Generating an endoreversible model in such a way, that the external exchanges are identical with those of an irreversible real running process.
- Reconstruction means: Generating model diagams from a given endoreversible model structure and chosen (cycle time dependent) reconstruction parameter diagrams (e.g., for testing the chosen endoreversible model by comparison with machine diagrams).

For elucidation, two very well-known endoreversible models are examplarily considered: the reversible Carnot process with Fourier heat leak as a first model parameter and a corresponding heat conductance as a second model parameter, and the Curzon–Ahlborn model, i.e., a reversible Carnot process with Newton heat conduction containing two internal temperatures as first model parameters and two heat conductances as second model parameters.

In the case of simulation, the model parameters—now called simulation parameters—depend on the machine diagrams of the real running engine which is simulated: for instance, for the first model the heat leak and the corresponding heat conductance are proportional to the reservoir-related entropy production, which follows from the machine diagrams.

In the case of reconstruction, the model parameters—now called reconstruction parameters—are *chosen* as functions of the cycle time by specifying the reconstruction parameter diagrams. These in connection with the endoreversible model structure provide the external heat exchanges in the form of the model diagrams. For instance, for the Curzon–Ahlborn model, the heat conductances are set constant and the intermediate temperatures are optimized for power output.

From a historical point of view, in the literature usually the reconstruction route has been taken, so that the connection between chosen endoreversible models and corresponding real running processes needs more elucidation which is the aim of this paper.

## Appendix A

### Appendix A.1. Non-Reversibility

From (35)${}_{3}$ follows

(A1) $$Q_{H}\left(\frac{\Lambda }{T_{H}}-\frac{1}{\Theta^{+}}\right)\;=\;Q_{L}\left(\frac{1}{\Theta^{-}}-\frac{\Lambda }{T_{L}}\right)\;⟶\;\alpha \left(\frac{1}{\Theta^{+}}-\frac{\Lambda }{T_{H}}\right)\;=\;\frac{1}{\Theta^{-}}-\frac{\Lambda }{T_{L}}$$

by use of (33)${}_{2}$. This results in

(A2) $$\Lambda \left(\frac{1}{T_{L}}-\frac{\alpha }{T_{H}}\right)\;=\;\frac{1}{\Theta^{-}}-\frac{\alpha }{\Theta^{+}}\;⟶\;\Lambda \;=\;\frac{\left(\alpha \Theta^{-}-\Theta^{+}\right)T_{H}T_{L}}{\left(\alpha T_{L}-T_{H}\right)\Theta^{+}\Theta^{-}}.$$

### Appendix A.2. Maximal Power (General)

From (53) follows

(A3) $$0\leq \frac{P}{\Omega /\Lambda }\;=\;\frac{T_{L}}{\tau (\eta )}\frac{\eta }{\left(\eta_{rev}-\eta \right)},\;⟹\;\left(\frac{P}{\Omega /\Lambda }\right){|}_{\eta =0}\;=\;0,\;\left(\frac{P}{\Omega /\Lambda }\right){|}_{\eta =\eta_{rev}}\;=\;0,$$

if

(A4) $$\lim_{\eta \to \eta_{rev}}\left[\tau \left(\eta \right)\left(\eta_{rev}-\eta \right)\right]\;\to \;\infty$$

is presupposed. From (A3)${}_{2}$ follows

(A5) $$\frac{d}{d\eta }\left(\frac{P}{\Omega /\Lambda }\right)\;=\;T_{L}\frac{\tau \left(\eta_{rev}-\eta \right)-\eta \left[\left(d\tau /d\eta \right)\left(\eta_{rev}-\eta \right)-\tau \right]}{{\left[\tau \left(\eta_{rev}-\eta \right)\right]}^{2}}\;≐\;0,$$

resulting in

(A6) $$\tau \eta_{rev}\;=\;\frac{d\tau }{d\eta }\eta \left(\eta_{rev}-\eta \right)\;⟹\;\frac{d\ln\tau }{d\eta }\;=\;\frac{\eta_{rev}}{\eta (\eta_{rev}-\eta )}.$$

An $\eta^{*}$ is sought for satisfying (A6)${}_{2}$

(A7) $$\frac{d}{d\eta }\ln\tau \left(\eta \right){|}_{\eta^{*}}\;=\;\frac{\eta_{rev}}{\eta^{*}\left(\eta_{rev}-\eta^{*}\right)}\;\neq \;\frac{1}{\eta^{*}},$$

determining the cycle time $\tau (\eta^{*})$ and the efficiency $\eta^{*}$ for which P/(Ω/Λ) is maximal. A comparison with (61)${}_{2}$ demonstrates that $P/(Q_{H})$ and P/(Ω/Λ) are maximal for different efficiencies.

### Appendix A.3. Power (Carnot Process with Fourier Heat Leak)

Starting with (77)

(A8) $$\lambda^{hl}\tau {\left(\frac{\eta_{rev}}{T_{L}}\right)}^{2}\;=\;-\frac{Q_{H}}{T_{H}}-\frac{Q_{L}}{T_{L}},$$

resulting by use of (49)${}_{3}$ in

(A9) $$\lambda^{hl}\tau \frac{T_{H}}{Q_{L}}{\left(\frac{\eta_{rev}}{T_{L}}\right)}^{2}\;=\;-\frac{Q_{H}}{Q_{L}}-\frac{T_{H}}{T_{L}}\;=\;\alpha -\alpha_{rev}\;=\;\lambda^{hl}\tau \frac{T_{H}}{Q_{L}}\frac{1}{T_{H}^{2}}{\left(\frac{T_{H}}{T_{L}}-1\right)}^{2}.$$

Using (41)${}_{2}$, this results in

(A10) $$\frac{Q_{L}}{\tau }\;=\;\frac{P}{1-\alpha }\;=\;\lambda^{hl}\frac{1}{T_{H}}\frac{{\left(\alpha_{rev}-1\right)}^{2}}{\alpha -\alpha_{rev}}$$

which is identical with (78).

### Appendix A.4. Reservoir-Related Entropy Production

Starting with (33)${}_{2}$ and (82),

(A11) $$\begin{array}{ccc} \alpha \;=\;-\frac{Q_{H}}{Q_{L}} & = & \frac{\lambda_{H}\left(T_{H}-T_{iH}\right)}{\lambda_{L}\left(T_{iL}-T_{L}\right)}\;=\;\frac{\lambda_{H}\left(T_{H}-\alpha T_{iL}\right)}{\lambda_{L}\left(T_{iL}-T_{L}\right)}, \end{array}$$

(A12) $$\begin{array}{ccc} \lambda_{L}\left(T_{iL}-T_{L}\right) & = & \lambda_{H}\left(T_{H}/\alpha -T_{iL}\right), \end{array}$$

(A13) $$\begin{array}{ccc} T_{iL}\left(\lambda_{L}+\lambda_{H}\right) & = & \lambda_{H}T_{H}/\alpha +\lambda_{L}T_{L}, \end{array}$$

(A14) $$\begin{array}{ccc} T_{iL} & = & \frac{\lambda_{H}T_{H}/\alpha +\lambda_{L}T_{L}}{\lambda_{L}+\lambda_{H}}. \end{array}$$

Consequently, the reservoir-related entropy production (83) becomes

(A15) $$\frac{\Omega }{\Lambda }\;=\;\frac{\lambda_{L}}{T_{H}}\tau \left(T_{L}-T_{iL}\right)\left(\alpha -\alpha_{rev}\right)\;=\;\frac{\lambda_{L}}{T_{H}}\tau \left(T_{L}-\frac{\lambda_{H}T_{H}/\alpha +\lambda_{L}T_{L}}{\lambda_{L}+\lambda_{H}}\right)\left(\alpha -\alpha_{rev}\right).$$

Inserting (49)${}_{4}$, this results in

(A16) $$\begin{array}{ccc} \frac{\Omega }{\Lambda }\;\; & = & \;\;\frac{\lambda_{L}}{\alpha_{rev}}\tau \left(1-\frac{\lambda_{H}\alpha_{rev}/\alpha +\lambda_{L}}{\lambda_{L}+\lambda_{H}}\right)\left(\alpha -\alpha_{rev}\right)=\frac{\lambda_{L}}{\alpha_{rev}}\tau \left(\frac{\lambda_{H}\left(1-\alpha_{rev}/\alpha \right)}{\lambda_{L}+\lambda_{H}}\right)\left(\alpha -\alpha_{rev}\right)=\; \end{array}$$

(A17) $$\begin{array}{ccc} & = & \tau \frac{\lambda_{L}\lambda_{H}}{\lambda_{L}+\lambda_{H}}\left(1-\frac{\alpha_{rev}}{\alpha }\right)\left(\frac{\alpha }{\alpha_{rev}}-1\right). \end{array}$$

### Appendix A.5. Work (Curzon–Ahlborn Model, Newton Heat Conduction)

Starting with (85), the work becomes, by the use of (A14), (80)${}_{2}$, and (49)${}_{4}$,

(A18) $$\begin{array}{ccc} -W\;=\;\lambda_{H}\tau \left(T_{H}-T_{iH}\right)\eta & = & \lambda_{H}\tau \left(T_{H}-\frac{\lambda_{H}T_{H}+\alpha \lambda_{L}T_{L}}{\lambda_{L}+\lambda_{H}}\right)\eta \;= \end{array}$$

(A19) $$\begin{array}{ccc} & = & \lambda_{H}\tau T_{H}\left(1-\frac{\lambda_{H}+\alpha \lambda_{L}/\alpha_{rev}}{\lambda_{L}+\lambda_{H}}\right)\eta . \end{array}$$

Taking (34)${}_{2}$ into account, the work becomes

(A20) $$\begin{array}{ccc} -W & = & \lambda_{H}\tau T_{H}\left(\frac{\lambda_{L}-\alpha \lambda_{L}\left(1-\eta_{rev}\right)}{\lambda_{L}+\lambda_{H}}\right)\eta \;=\;\lambda_{L}\lambda_{H}\tau T_{H}\left(\frac{1-\left(1-\eta_{rev}\right)/\left(1-\eta \right)}{\lambda_{L}+\lambda_{H}}\right)\eta =\; \end{array}$$

(A21) $$\begin{array}{ccc} & = & \tau \frac{\lambda_{L}\lambda_{H}}{\lambda_{L}+\lambda_{H}}T_{H}\left(1-\frac{1-\eta_{rev}}{1-\eta }\right)\eta \;=\;\tau \frac{\lambda_{L}\lambda_{H}}{\lambda_{L}+\lambda_{H}}T_{H}\frac{\eta_{rev}-\eta }{1-\eta }\eta , \end{array}$$

resulting in (85).
